# Population screening and transmission experiments indicate paramyxid-microsporidian co-infection in *Echinogammarus marinus* represents a non-hyperparasitic relationship between specific parasite strains

**DOI:** 10.1038/s41598-018-22276-y

**Published:** 2018-03-16

**Authors:** Yasmin Guler, Stephen Short, Amaia Green Etxabe, Peter Kille, Alex T. Ford

**Affiliations:** 1 Institute of Marine Sciences, School of Biological Sciences, University of Portsmouth, Ferry Road, Portsmouth, Hampshire, PO4 9LY UK; 2 Cardiff School of Biosciences, Sir Martin Evans Building, Museum Avenue, Cardiff, CF10 3AT UK; 3 Centre for Ecology and Hydrology, Maclean Building, Benson Lane, Wallingford, Oxfordshire OX10 8BB UK

## Abstract

Phylogenetically distant parasites often infect the same host. Indeed, co-infections can occur at levels greater than expected by chance and are sometimes hyperparasitic. The amphipod *Echinogammarus marinus* presents high levels of co-infection by two intracellular and vertically transmitted parasites, a paramyxid (*Paramarteilia* sp. *Em*) and a microsporidian strain (*Dictyocoela duebenum Em*). This co-infection may be hyperparasitic and result from an exploitative ‘hitchhiking’ or a symbiotic relationship between the parasites. However, the best-studied amphipod species are often collected from contaminated environments and may be immune-compromised. Immune-challenged animals frequently present co-infections and contaminant-exposed amphipods present significantly higher levels of microsporidian infection. This suggests the co-infections in *E. marinus* may result from contaminant-associated compromised immunity. Inconsistent with hyperparasitism, we find that artificial infections transmit *Paramarteilia* without microsporidian. Our population surveys reveal the co-infection relationship is geographically widespread but find only chance co-infection between the *Paramarteilia* and another species of microsporidian, *Dictyocoela berillonum*. Furthermore, we identify a haplotype of the *Paramarteilia* that presents no co-infection, even in populations with otherwise high co-infection levels. Overall, our results do not support the compromised-immunity hypothesis but rather that the co-infection of *E. marinus*, although non-hyperparasitic, results from a relationship between specific *Paramarteilia* and *Dictyocoela duebenum* strains.

## Introduction

Invertebrates are infected by a varied array of intracellular parasites. These include both the Paramyxida (Rhizaria, Acetosporea), an order of parasitic protists commonly referred to as paramyxeans^1,2^ and Microsporidia, a diverse phylum that infect hosts from all major taxa^3^. Species from both parasite groups have adopted a variety of life cycle strategies. Some are horizontally transmitted pathogens that cause substantial mortalities in economically important species^2,3^ while others are vertically transmitted and have less destructive impacts on their hosts. For example, some microsporidians subvert the amphipod sex determination mechanism and convert males into females^4,5^. This feminising capacity appears to have evolved independently in several microsporidian lineages, including strains of *Nosema granulosis* and *Dictyocoela duebenum*^4–6^. This strategy is thought to have evolved to allow parasites to maximise their transmission via the host’s progeny by converting males into reproductive females^7^. It has been recently confirmed that paramyxid parasites can also feminise amphipods, specifically, the amphipod *Orchestia gammarellus* infected with *Paramarteilia orchestiae* produce significantly more female and intersex offspring^8^.

As well as having manipulative associations with their hosts, it has become clear that paramyxid and microsporidian parasites have a range of relationships with each other when infecting the same host. One such relationship is a clear case of hyperparasitism, a scenario in which a parasite’s host is itself a parasite. Specifically, microsporidian spores infect multiple *Marteilia* species of paramyxid that, in turn, infect oysters^9^, mussels^10^ and cockles^11,12^. The advantage gained by this hyperparasitism is still unclear but the strategy is thought to aid transmission of the microsporidian into an intermediate crustacean host^11^.

In addition to hyperparatism, there appear to be less intimate microsporidian-paramyxid co-infection relationships. These can be chance co-infections, as seems to be the case for the microsporidian *Dictyocoela berillonum* and a paramyxid species (currently termed *Paramarteilia* sp. *Em*) that infect the amphipod *Echinogammarus marinus*^13^. In addition, there are co-infections that occur at levels greater than would be expected by chance, as is the case for a *Paramarteilia* species and the microsporidian *Dictyocoela cavimanum* in the amphipod *Orchestia aestuarensis*^8^ and also *Dictyocoela duebenum Em* (from here on referred to as *D. duebenum*) and *Paramarteilia* sp. *Em* (from here on referred to as *Paramarteilia*) in *E. marinus*^13^. Although co-infection predominates in *E. marinus*, solitary *Paramarteilia* infections are also found. Such solitary infections are reasonable given microscopic analysis of *E. marinus* tissue infected with *D. duebenum* and *Paramarteilia*. This revealed that the microsporidian does not seem to hyperparasitise the paramyxid, rather the parasites form separate infections, with the paramyxid and microsporidian sometimes being found within the cytoplasm of the same host cell^11^. The lack of a hyperparasitic connection between the co-infecting parasites is entirely consistent with finding cases of *Paramarteilia* infection without *D. duebenum*.

When parasites possess similar life cycles and infect the same host it can favour the evolution of a ‘hitchhiking’ strategy^14^. Given the associations between paramyxids, microsporidians and amphipod feminisation^4,8^, it is possible that one of these parasites is a more efficient feminiser in any given host species or environment. This scenario would allow the hitchhiker to benefit from the feminising capacity of its co-infecting parasite. In fact, experimentally determined feminisation capacities has led to this hypothesis in the case of a co-infection between a *Paramarteilia* sp. and *D. cavimanum* in *O. aestuarensis*^8^. It is also possible that co-infections are mutually beneficial, as it is conceivable that the combined efforts of the microsporidian and paramyxid more effectively feminise their host. Co-infection may also be related to the capacity of one or other parasite to manipulate host immunity^15^, leading to immune suppression that is exploited by the other parasite and increasing the likelihood of co-infection. For example, heminth-mediated host immune suppression leads to co-infection with the malaria parasite^16^ and a similar supression mechanism could help explain the avirulence associated with paramyxid and microsporidian infection in *E. marinus*. As suggested for feminisation, it is possible that both parasites contribute to immune suppression and the co-infection reflects a symbiotic relationship. Feminisation and immune suppression, or potentially some combination, rely on one or both parasites exploiting the host-manipulating capacity of its co-infecting parasite. Therefore, in such scenarios, co-infection would reflect a relationship that has evolved between particular parasites.

However, despite the plausibility that hitchhiking or symbiotic strategies underlie co-infection patterns, they are not the only potential explanation. Exposure of amphipods to contaminants results in significant increases in infection by vertically transmitted microsporidia^17,18^. In addition, theoretical predictions and genetic evidence suggests that vertically transmitted Microsporidia utilise horizontal transmission to some extent^19,20^. When put together, these findings raise the possibility that co-infection patterns occur due to an increased likelihood that contaminant-exposed animals have impaired immunity and will contract any parasites with a sufficient prevalence in the population. This could be because of increased vertical and/or horizontal transmission efficiencies in compromised animals. Horizontal transmission via cannibalism can be an important route of infection in *Gammarus* amphipods^21^ and a deficient immune system may increase its efficiency. An association between host immune deficiency and parasite co-infection is supported by work in other arthropod species. Bumblebees present more frequent co-infections with various strains of the trypanosome *Crithidia bombi* when immune compromised^22^. When considering an immune deficiency hypothesis in the case of *E. marinus*, it is worth noting that the single population found to be co-infected were collected from a heavily contaminated site^23,24^. Such a hypothesis would mean the observed co-infection is a by-product of contaminant exposure and not the result of an evolved relationship between specific parasites.

Although limited screening has suggested the presence of *Paramarteilia* in *E. marinus* populations that are also infected with the closely related microsporidian *D. berillonum*, initial results indicate a lack of co-infection^13^. If confirmed, the absence of co-infection would argue against the compromised-immunity hypothesis. Furthermore, it was recently found that the amphipod *O. aestuarensis* collected from the same population hosts multiple *Paramarteilia sp*. haplotypes^8^, raising the possibility that a variety of *Paramarteilia* species/strains possessing divergent transmission strategies may infect animals in the same population. The existence of distinct co-infecting and solitary-infecting *Paramarteilia* strains in the same populations would strongly support the hypothesis that the co-infections are a result of an evolved relationship between specific parasites. In this study we explore parasitic transmission and characterise *Paramarteilia* and *Dictyocoela* infection in *E. marinus* populations and discuss these results in the context of hyperparasitism and our understanding of co-infection in *E. marinus*.

## Results

### Unlike *Paramarteilia* - *D. duebenum* co-infection, *Paramarteilia - D. berillonum* co-infection occurs at coincidental levels

**Figure 1:**
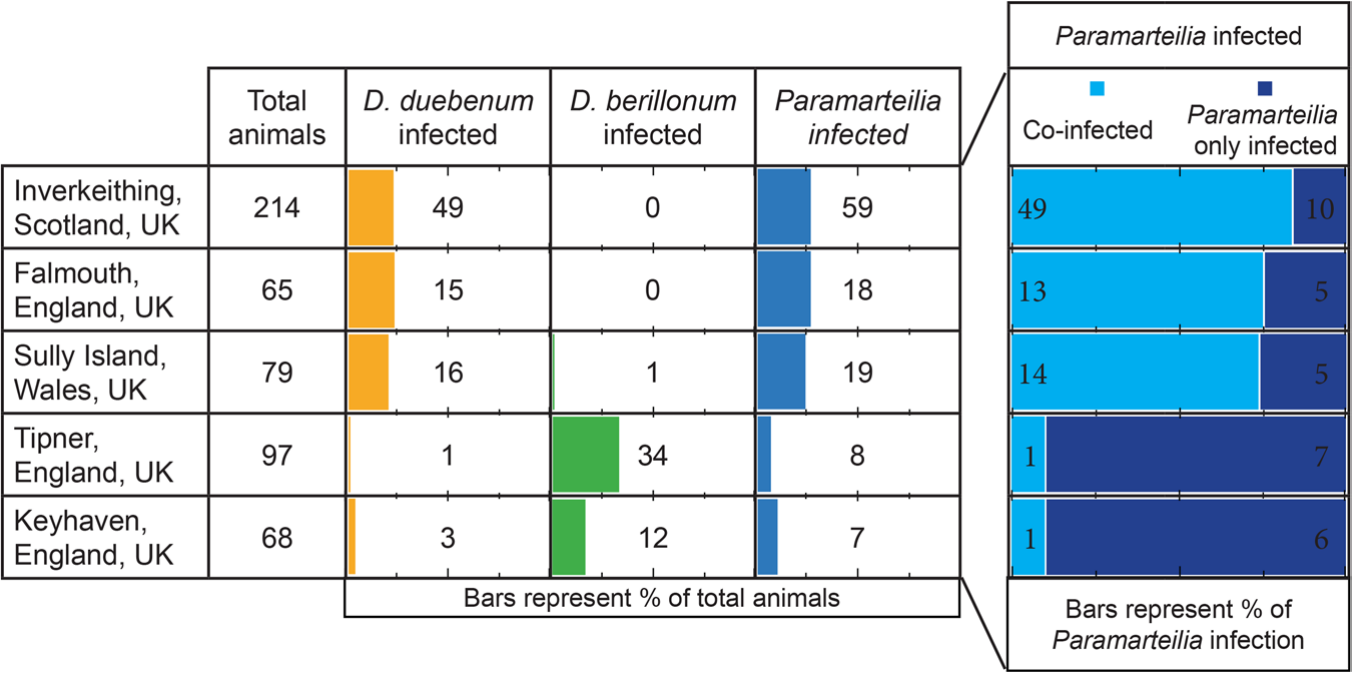
Prevalence of *Paramarteilia* and *D. duebenum* infection of *Echinogammarus marinus* at various sites around the UK.

The five surveyed populations presented two types of infection profile. At three sites (Inverkeithing, Sully Island and Falmouth) infections are dominated by *Paramarteilia* and *D. duebenum*, while the other two (Tipner and Keyhaven), are dominated with *Paramarteilia* and *D. berillonum* infections (Fig. 1). The distribution of infections among the various sexual phenotypes of *E. marinus* present at Inverkeithing was comparable to those previous described^13^. Given the prevalence of both parasites at the three *Paramarteilia* - *D. duebenum* dominated sites, the occurrence of co-infection was greater than expected by chance. The frequency of *D. duebenum* infection in the population at Inverkeithing was found to be 23% (Fig. 1), assuming no bias towards co-infection, it would be reasonable to expect 14 of 59 (0.23 × 59) animals infected with *Paramarteilia* to be co-infected with *D. duebenum*. However, 49 cases of co-infection were observed and 10 cases of infection with only *Paramarteilia*, a result that is highly significant (Chi-Square; χ^2^ = 39.4, df = 1 P < 0.0001) and consistent with that found in a previous survey^13^. The level of *Paramarteilia* and *D. duebenum* co-infection was also significant at Falmouth (χ^2^ = 7.1, df = 1 P = 0.0076), and Sully Island (χ^2^ = 8.55, df = 1 P = 0.0035). In contrast, the observed co-infection between *Paramarteilia* and *D. berillonum* appears to be coincidental at Tipner (χ^2^ = 0.3, df = 1 P = 0.5637) and Keyhaven (χ^2^ = 0.0, df = 1 P = 1.0000). The co-infection levels are still consistent with chance when the infection numbers for both Tipner and Keyhaven are combined (χ^2^ = 0.208, df = 1 P = 0.6481).

The three sites dominated by *Paramarteilia* and *D. duebenum* infection have very similar prevalence of *D. duebenum*, (23%, 23% and 20%) and *Paramarteilia* (28%, 28% and 24%) (Fig. 1). Across these three sites, 79% of *Paramarteilia* infected animals are co-infected with *D. duebenum*. The prevalence of *D. berillonum* across the two sites dominated with *Paramarteilia* and *D. berillonum* is more variable (35% and 18%) and has an overall prevalence of ~28%. If the same level of co-infection occurred between *Paramarteilia* and *D. berillonum* at Tipner and Keyhaven, it would be reasonable to expect 12 (15 × 0.791) of the 15 paramyxid infected animals to be co-infected with *D. berillonum*. However, we see just a single case of *Paramarteilia* and *D. berillonum* co-infection, a discrepancy that is significant (χ^2^ = 13.575, df = 1 P = 0.0002).

### Vertical transmission efficiency of *Paramarteilia* is unaffected by co-infection

**Figure 2:**
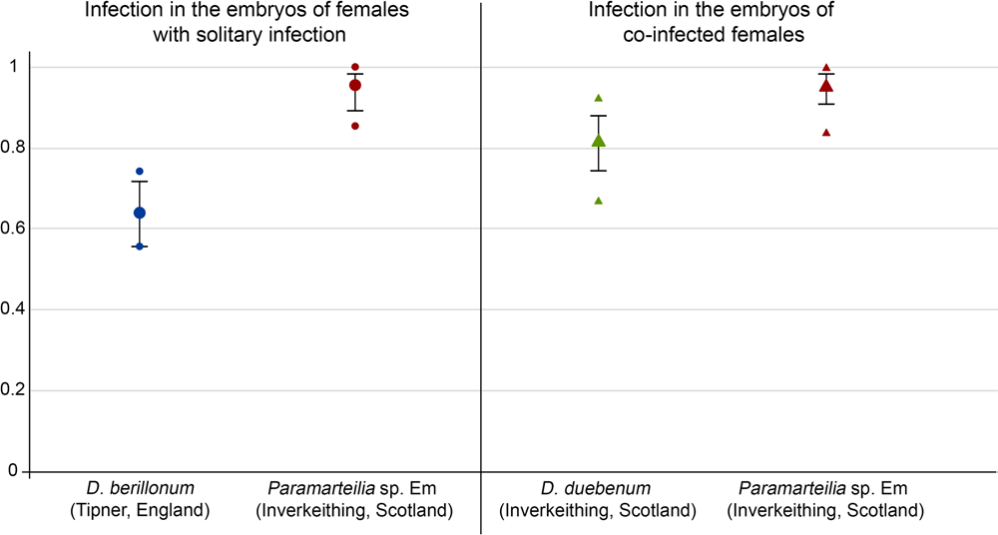
Proportion of embryos infected by *D. berillonum*, *D. duebenum* and *Paramarteilia* taken from broods of solitary (circles) and co-infected mothers (triangles). Seven broods were tested per infection group. Mean of all seven broods is shown (larger markers) for each infection group. Error bars indicate 95% binomial confidence intervals. The small markers above and below the mean represent the broods with the highest and lowest proportions of infected embryos. Note: no solitary infected *D. duebenum* category is possible due to a lack of animals with solitary *D. duebenum* infection.

An initial PCR screen performed using DNA isolated from pooled broods removed from infected mothers indicate that *Paramarteilia*, *D. duebenum* and *D. berillonum* transmit their infection vertically to their offspring (data not shown). The vertical transmission efficiency of parasites was then assessed in more detail for the three parasites in various infection types: *Paramarteilia* and *D. berillonum* in the broods of females presenting solitary infections, as well as *Paramarteilia* and *D. duebenum* in broods of co-infected females. Seven broods were screened for each infection type and revealed efficient vertical transmission for all parasites, while the control broods (taken from uninfected gravid females) presented no evidence of infection (Fig. 2). The differences in transmission rate (i.e. the average proportion of infected embryos) between the four infection types were significant (Kruskal–Wallis test: χ$\frac{2}{3}$ = 20.47; P = 0.00014). Post-hoc pairwise multiple comparison tests were conducted to determine which infection-type pairs are significantly different. The transmission efficiency of *D. berillonum* was significantly lower than *Paramarteilia* in solitary infections (Dunn’s test with P-value adjusted by the Benjamini-Hochberg FDR method, P = 0.0004) or *Paramarteilia* when co-infected with *D. duebenum* (Dunn’s test with P-value adjusted by the Benjamini-Hochberg FDR method, P = 0.0012). Of the microsporidians, *D. duebenum* (co-infected with *Paramarteilia*) presented a higher transmission efficiency than *D. berillonum* (using broods isolated from females with solitary infections) but this difference was not significant (Dunn’s test with P-value adjusted by the Benjamini-Hochberg FDR method, P = 0.1449). The highest transmission efficiency was observed in *Paramarteilia* only infections, with slightly reduced *Paramarteilia* transmission efficiency observed in the broods of females co-infected with *Paramarteilia* and *D. duebenum*. However, this difference is not significant (Dunn’s test with P-value adjusted by the Benjamini-Hochberg FDR method, P = 0.6592).

### *Paramarteilia* transmission following injection of co-infected tissue is inconsistent with hyperparasitism

**Figure 3:**
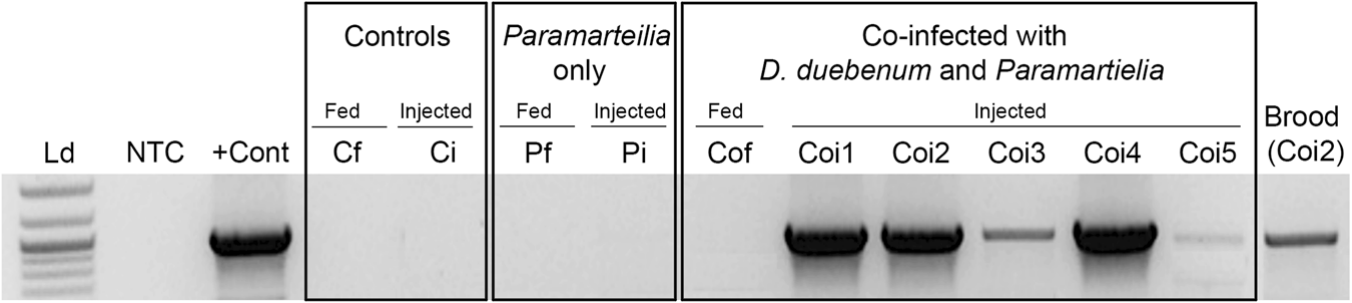
Screen for the presence of *Paramarteilia in Echinogammarus marinus* after feeding or injection by *Paramarteilia* only infected, *Paramarteilia* - *Dictyocoela duebenum* co-infected or uninfected *E. marinus* tissue. Ld – DNA ladder, NTC – No Template Control, +Cont – *Paramarteilia* positive control sample. Controls = Cf - Control fed with infected tissue (n = 7), Ci - Control injected with uninfected tissue (n = 4). *Paramarteilia* only = Pf - Fed with tissue infected by *Paramarteilia* only (n = 7), Pi – Injected with tissue infected by *Paramarteilia* only (n = 4). Co-infected by *D. duebenum* and *Paramartielia* = Cof - Co-infection fed (n = 11). Coi1-Coi5 – Represents co-infection injected individuals. Brood (Coi2) - Brood harvested from an ovigerous female (Coi2) four months after injection by co-infected tissue.

Individuals (ten male and ten female *E. marinus* per group) from a population at Langstone Harbour, UK were fed or injected with infected tissue dissected out of individuals collected from the Inverkeithing population. The control groups were fed and injected with tissue from uninfected individuals taken from the Langstone Harbour population. This source population was chosen because a survey revealed no prevalence of *Paramarteilia* or *D. duebenum* (data not shown). The six experimental groups consisted of animals fed and injected with: control tissue, tissue infected with *Paramarteilia* only and tissue co-infected with *Paramarteilia* and *D. duebenum*. Following a four-month incubation period, the surviving animals were sacrificed and a PCR screen was performed. Evidence of artificial horizontal transmission was only observed in the surviving animals injected with tissue co-infected with *Paramarteilia* and *D. duebenum* (Fig. 3, Coi1-Coi5). Following the incubation, a screen of pooled DNA from the late stage embryos of one of the surviving females revealed a *Paramarteilia* infection (Fig. 3, Brood Coi2). Although only semi-quantitative, the band intensities suggest the paramyxid burden was higher in the females (Fig. 3, lanes Coi1, 2 and 4) than the males (Fig. 3, lanes Coi3 and 5). *D. duebenum* showed no artificial horizontal transmission in any of the fed or injected groups.

### *Paramarteilia* haplotype 117A presents no co-infection, even in populations with otherwise high co-infection levels

We sequenced a 980 bp fragment of the *Paramarteilia* 18S ribosomsal RNA gene from 48 animals across the five collection sites and found evidence for two haplotypes. The first is identical to the previously sequenced *Paramarteilia* (JQ673484) isolated from infected *E. marinus* collected from Inverkeithing, Scotland^13^. The second haplotype has an identical 18S rRNA gene sequence with the exception of a transition from G to A at position 117. The 117A haplotype sequence, previously termed ‘*Oa*1’ (KY056194) has been found following population surveys of *O. gammarellus* and *O. aestuarensis* in France and the UK^8^. The results reveal that the 117G haplotype is present at all 5 collections sites, while the 117A haplotype is present in at least three (Table 1). The animals selected for the screen composed of 24 solitary-infected and 24 co-infected animals. All *Paramarteilia* - *D. duebenum* co-infected animals were infected with the 117G haplotype whereas the 24 solitary-infected animals were infected equally with 117G and 117A haplotypes (Table 1). Two of the sample sites (Sully Island and Inverkeithing), which have high levels of *D. duebenum* and present high levels of *Paramarteilia* - *D. duebenum* co-infection, also host the 117A haplotype (Table 1). Given the 117A haplotype prevalence in the samples surveyed from these sites (9/29 = 31%), and assuming a random haplotype distribution, it is reasonable to expect 5 (15 × 0.31) of the 15 co-infected animals from these sites to harbour the 117A haplotype. However, no instances of *Paramarteilia* 117A - *D. duebenum* co-infection was observed at these sites, a finding that is significant (Fisher’s exact test, P = 0.0407).

**Table 1:** Characterisation of *Paramarteilia* haplotypes present in *Echinogammarus marinus*. *Also termed *Oa*1^8^.

Haplotype	Total (of 48 haplotyped animals)	Co-infected with *D. duebenum*	*Paramarteillia* only infected	Sites	Associated phenotypes
117A*	12	0	12	Inverkeithing, Scotland; Sully Island, Wales; Tipner, England	NM, NF
117G	36	24	12	Found at all collection sites	NM, NF, EIM, IF

## Discussion

Consistent with the idea that both *Paramarteilia* and *D. duebenum* are predominantly vertically transmitted, no horizontal transmission was observed in animals fed infected tissue and high levels of parasite infection were found in the embryos of infected mothers. Furthermore, finding comparable levels of vertical transmission in co-infected and *Paramarteilia*-only infected animals suggests the presence of *D. duebenum* does not meaningfully change the transmission efficiency of the *Paramarteilia* when co-infecting with *D. duebenum*. The artificial infection by tissue injection successfully transmitted the *Paramarteilia* to recipient animals but failed to transmit the co-infecting *D. duebenum*. A similar finding was reported following the transplantation of *Paramarteilia* - microsporidian co-infected tissue into *O. aestuarensis*^25^. In that case, ultrastructural analysis following transplantation of infected tissue revealed that only *Paramarteilia* cells were present in the recipient animals. It is not clear why only the *Paramarteilia* infection can be successfully transmitted using this technique but this finding is consistent with the *D. duebenum* existing independently of the *Paramarteilia* within the host’s cells. It is reasonable to assume that if *D. duebenum* hyperparasitises the *Paramarteilia*, it would have successfully transferred to the recipient amphipod within the cells of its successfully transplanted host *Paramarteilia*. This finding is also consistent with briefly described electron microscopy analysis that suggests the parasites form two separate infections within the same individual^11^. It is possible that there is some level of hyperparasitism but that the parasitised *Paramarteilia* cells don’t survive the transfer process or don’t proliferate efficiently within the tissue of the recipient animal. This could be possible if the microsporidian inhibits the normal function of the paramyxid in any hyperparasitised cells. However, in a comprehensively described case of hyperparasitism, no inhibition of spore formation was observed for the paramyxid *Marteilia cochillia* when hyperparasitised by the microsporidian *Hyperspora aquatica*^11^. Overall, the experimental evidence points to the relationship between specific strains of *Paramarteilia*, and *D. duebenum* being a case of non-hyperparasitic co-infection, a type of paramyxid-microsporidian relationship distinct from the clear hyperparasitism described in a range of bivalves^9–12^.

Our survey found evidence for two *Paramarteilia* haplotypes, that for convenience are being termed 117G and 117A. *Paramarteilia* 117G and *D. duebenum* was found to infect animals from all five populations surveyed, suggesting that amphipod infection by these parasites is widespread around the UK. Consistent with our findings, a higher than coincidental rate of *Paramarteilia* - *D. duebenum* co-infection has been previously reported for the Inverkeithing collection site^13^. Analysis of individuals sampled from Falmouth and Cardiff revealed that the co-infection relationship between *Paramarteilia* - *D. duebenum* is not restricted to a single *E. marinus* population. The widespread nature of the co-infecting relationship is, in itself, arguably consistent with either the existence of a relationship between parasites or the hypothesis of co-infection resulting from contaminant-induced immune deficiency. However, where information is available, the collection sites have distinct contamination profiles^23,26–28^, so if the *E. marinus* at the three sites dominated by co-infection are immune compromised, it is not the result of a specific contaminant at Inverkeithing. Of course, it is possible that the stress caused by the distinct cocktail of various metals, polychlorinated biphenyls (PCBs) and pentachlorophenols (PCPs) at each site^23,26–28^ induces comparable levels of immune deficiency. However, contaminant surveys close to the Tipner site, which presents coincidental rates of co-infection between *Paramarteilia* and *D. berillonum*, reveal considerable levels of PCPs and metal pollution^27,28^. Although no site will have an identical contamination profile, it could be argued that if contaminant-induced immune deficiency is occurring at the three sites presenting higher than coincidental rates of co-infection, it could also be expected between *Paramarteilia* and *D. berillonum* at the Tipner site.

The observed co-infection between *Paramarteilia* and *D. berillonum* are consistent with chance and suggests that the striking co-infection relationship found in UK *E. marinus* populations is limited to *Paramarteilia* and *D. duebenum*. Given that the reference strain of *D. duebenum* (Genbank: AF397404) infects the amphipod *Gammarus duebeni* without a paramyxid^4^, it appears that co-infection is restricted to a specific strain of *D. duebenum* (Genbank: JQ673483). Consistent with this hypothesis, the survey of the Tipner population revealed a single case of infection with strain of *D. duebenum* (Genbank: JQ673482) that is very closely related to the reference strain (Genbank: AF397404) and no co-infection was found in this single example. Furthermore, the lack of co-infection between *D. duebenum* and the 117A haplotype of *Paramarteilia* suggests the co-infecting relationship occurs between specific strains of both *Paramarteilia* and *D. duebenum*, especially as high levels of *D. duebenum* are found in two of the populations hosting the 117A haplotype. Critically, the observation of chance co-infection between *Paramarteilia* and *D. berillonum* and, more strikingly, the different levels of co-infection observed for the 117G and 117A haplotypes of *Paramarteilia* argues strongly against the immune deficiency hypothesis. Presumably, animals hosting these haplotypes collected from the same location have had comparable exposure to any anthropogenic contamination. Therefore, if the co-infection observed at Inverkeithing and Cardiff were the result of contamination-induced immune deficiency, it would be reasonable to expect equal numbers of co-infections involving both 117A and 117G haplotypes across these sites. It is also worth noting that the differential co-infection patterns observed for these haplotypes suggests that closely related paramyxids can possess notably divergent strategies/life cycles, as is the case for microsporidia^4^. Furthermore, a recent screen of *O. gammarellus* and *O. aestuarensis* amphipods identified a *Paramarteilia* haplotype identical to 117A (termed *Oa*1) that infects both amphipod species^8^. The observation that this haplotype is also capable of infecting *E. marinus* suggests this paramyxid has multiple hosts.

The infection patterns across the five sites strongly suggest that the observed *Paramarteilia* - *D. duebenum* co-infection reflects a relationship that has evolved between specific parasite strains. However, the strategy that underlies this relationship remains unclear. Our survey revealed solitary *D. duebenum* infections in *E. marinus* are rare, with at least one case being due to infection by the distinct *D. duebenum* reference strain that is not associated with co-infection^4^. By contrast, solitary *Paramarteilia* infections are found at consistent levels at every site, indeed, the 117G haplotype is found as a solitary infector in populations with a high prevalence of *D. duebenum* and co-infection. This suggests that the *D. duebenum* is more dependent on co-infection than the *Paramarteilia* for successful transmission in *E. marinus* and may be ‘hitch-hiking’. The microsporidian may be exploiting a potential feminisation capacity of the paramyxid, as has been recently suggested for *Paramarteilia* - *D. cavimanum* co-infection in *O. aestuarensis*^8^ and/or enhancing its transmission by taking advantage of the paramyxid’s ability to manipulate host immunity, a scenario observed in cases of co-infection by other parasite groups^16^. It may be noteworthy that of the 48 selected for haplotype analysis, 9 presented an intersex phenotype and none were infected with the *Paramarteilia* 117A haplotype. Such a finding supports a recent suggestion^8^ that non-feminising species or strains of *Paramarteilia* may also occur in amphipods. The capacity to produce *Paramarteilia*-only infected animals by injecting co-infected tissue will, combined with advances in *E. marinus* husbandry, increase the viability of breeding experiments to investigate the manipulative capabilities of the *Paramarteilia* 117G haplotype, as well as the wider investigation of sex determination in amphipods^29^.

The capacity of paramyxids and microsporidians to decimate valuable cultured and wild animal populations^2,3^ makes it important to understand their lifecycles. Considerable insights into the basic biology of paramyxid and microsporidian virulence could be gained by comparing the molecular biology of the avirulent species (such and *Paramarteilia* and *D. duebenum* in amphipods) with virulent species that induce severe pathologies. Furthermore, it appears that paramyxids and microsporidians have a range of relationships, from chance co-infections and co-infections at higher than coincidental rates^8,13^, through to hyperparasitism of paramyxid cells by microsporidians^11^. A transcriptomic and genomic comparison of closely related co-infecting and non co-infecting strains within the same host could answer questions such as: do co-infecting parasites modulate their transcriptomic output to accommodate the presence of their co-infecting partner? Has co-infection, which results in both parasites occupying the cytoplasm of the same host cells, led to horizontal transfer of functionally important genes between the parasites? Such gene transfer may lead to one or both parasites becoming largely or completely dependent on its co-infecting partner for successful transmission. Indeed, the co-infection patterns suggest this might be the case for co-infecting strain of *D. duebenum* in *E. marinus*. A molecular comparison between the *Paramarteilia* 117G haplotype with the solitary infecting 117A haplotype, as well as the co-infecting *D*. *duebenum* strain with the *D*. *duebenum* reference strain, ought to reveal insights into molecular dimension of such co-infection relationships. The rapidly developing transcriptomic resources available for infected and uninfected *E. marinus* are already being utilised to develop reproductive biomarkers^30,31^. The utilisation of these resources in conjunction with the knowledge of parasite haplotypes produced by this study promises to expand our understanding of these interesting and economically critical parasites.

## Methods

### Sampling, dissection and DNA isolation

*E. marinus* were collected from beneath seaweed and rocks in the intertidal zone during low tide in Inverkeithing Scotland (UK) (56°1′38′′N 3°23′37′′W) in March 2012 and a screen for microsporidian and paramyxid parasites was conducted. Animals were anaesthetised in clove oil (0.4 µl/ml) and the sexual phenotype was determined (female, intersex female, male or intersex male). Additional samples of *E. marinus* were collected from Inverkeithing, Scotland, Sully Island, Wales, UK (51°23′ 48′′N 3°11′56′′W), Mylor Creek, Falmouth, UK (50°11′0′′N 5°4′30′′W), Tipner, Portsmouth, UK (50°49′ 39′′N 1°5′41′′W) and Keyhaven, Lymington, UK (50°43′ 13′′N 1°33′58′′W) between March 2014 and March 2015. Muscle and nervous tissue from adults was then harvested, and genomic DNA was purified using the DNeasy Blood and Tissue Kit (Qiagen, Hilden, Germany) following the manufacturer’s protocol, and quantified via spectrophotometry (NanoDrop ND-1000).

### Parasite screening

The DNA samples were screened for microsporidian and paramyxid parasite infection as previously described^4,13,32–34^. Briefly summarised, DNA quality was assessed by amplifying a 674 bp region of the host 18S ribosomal RNA gene using primers 1073 F (5′-CGGGGGGAGTATGGTTGC-3′) and 18SR (5′-TGATCCTTCTGCAGGTTCACCTAC-3′). The PCR was performed for 32 cycles using an annealing temperature of 60 °C and a 45 second extension time in a 25 µl volume containing 40 ng of template, 1 U of Taq polymerase (Promega, UK) and 2.0 mM MgCl_2_. Screening for Microsporidia was performed using general Microsporidia primers V1f (5′-CACCAGGTTGATTCTGCCTGAC-3′) and 1492 R (5′-GGTTACCTTGTTACGACTT-3′) that amplify a ~1.2 kb region of the microsporidian 16 S ribosomal RNA gene. The PCR was performed for 35 cycles with an annealing temperature of 58 °C and a 1 minute 45 second extension time in a 25 µl volume containing 40 ng of template, 1 U of Taq polymerase and 1.25 mM MgCl_2_. Samples positive for microsporidian infection were further screened using a primer sets specific for the microsporidian species *Dictyocoela berillonum*: V1f (see above) and BMR (5′-GATTTCTCTTCCGCAATACAGA-3′) and *Dictyocoela duebenum*: V1f and DMR (5′-GATTTCTCTTCCGCAATACCAAT-3′). The PCR was performed for 35 cycles using an annealing temperature of 58 °C and a 1 minute 30 second extension time in a 25 µl volume containing 40 ng of template, 1 U of Taq polymerase and 1.25 mM MgCl_2_. Samples were also screened for paramyxid parasite infection using primers JIparaF3 (5′-GATCAACGGGAGCGGT-3′) and JIparaR3 (5′-GCCCATCGGCAGAGGTAT-3′) which amplify a 391 bp fragment of the paramyxid 18S rRNA gene^4^. Various infected individuals were selected for haplotype analysis. Sequences for haplotype analysis were generated by PCR using the primer set ParaJI_F1 (5′-GGACCATTGCTGAGACTAAA-3′) and ParaJI_R1 (5′-GAGTTCAGAGAAACAGTTG-3′), which amplify a 980 bp fragment^4^. The PCR was performed for 35 cycles using an annealing temperature of 50 °C and an extension time of 1 minute in a 25 µl volume containing 40 ng of template, 1 U of Taq polymerase and 1.25 mM MgCl_2_. The amplified PCR product was purified using the QIAquick-spin PCR purification kit (Qiagen, UK) and sequenced (Eurofins MGW Operon, Germany).

### Parasite transmission

Ovigerous *E. marinus* females were taken from Langstone Harbour, Portsmouth, UK (50°47′23′′N 1°02′31′′W), and Inverkeithing, Scotland, UK (56°1′ 38′′N 3°23′37′′W) and DNA was extracted (DNAeasy Blood and Tissue Kit, Qiagen) from both adult tissues and any associated broods. The DNA of each egg or embryo was extracted individually and included an additional RNAse H (New England Biolabs, UK) step for 10 minutes. To establish transmission efficiency these samples (along with suitable uninfected controls) were screened for microsporidian and paramyxid infection using PCR in conjunction with the JIparaF3 - JIparaR3 and V1f - DMR primers to detect the presence of *Paramarteilia* and *Dictyocoela duebenum* respectively (as described above).

### Artificial infection

Specimens from Inverkeithing were anaesthetised by immersion in a clove oil solution (0.4 μl/mL of seawater) and the head, gut, and hepatopancreas removed. The body tissue was cut laterally and half the animal was stored at 4 °C in a 1.5 mL micro centrifuge tube containing 1 mL of seawater. DNA was then extracted from the gonadal and muscle tissue from the remaining half using the Phire® Animal Tissue Direct PCR Kit (Thermo Scientific, UK) using the manufacturer’s guidelines and subsequently screened for *Paramarteilia* and *Dictyocoela duebenum* infection using the PCR screen described above. The stored *E. marinus* tissue from Inverkeithing belonging to animals found to be co-infected by *Paramarteilia* and *Dictyocoela duebenum* were used in the attempt to infect Langstone Harbour *E. marinus* either by feeding or injecting. The fed group were starved for 7 days prior to being fed infected tissue. For the injected group, infected muscle and gonadal tissue was homogenised briefly in 1.5 mL micro centrifuge tube using a sterilised plastic pestle before recipient amphipods were inoculated by collecting ~10 μl tissue on the end of a syringe needle (26 s ga needle from a Hamilton® 700 Series Syringe, Sigma-Aldrich, UK) and injecting the homogenate between the 4th and 5th pereon. Animals in the control groups were fed and injected with tissue removed from PCR screened *E. marinus* taken from the Langstone Harbour (50°47′ 38′′N 1°1′57′′W) population, chosen because a survey revealed no prevalence of *Paramarteilia* or *D. duebenum* (data not shown). Ten males and ten females from the Langstone Harbour *E. marinus* population were infected per group. After four months, surviving animals were anesthetised in clove oil (0.4 μl/mL of seawater) and assessed. Muscle tissue, gonadal tissue and any embryos present were then dissected/removed before DNA was isolated (DNAeasy Blood and Tissue Kit, Qiagen) and used to perform a PCR screen for the presence of *Paramarteilia* and *Dictyocoela duebenum* as described above using the ParaJI_F1 and ParaJI_R1 primers.
